# RNAi screening for modulators of an osmo-sensitive gene response to extracellular matrix damage reveals negative feedback and interactions with translation inhibition

**DOI:** 10.1371/journal.pone.0285328

**Published:** 2023-05-08

**Authors:** Luke M. Chandler, Michael Rodriguez, Keith P. Choe

**Affiliations:** Department of Biology and Genetics Institute, University of Florida, Gainesville, FL, United States of America; Baylor University, UNITED STATES

## Abstract

In epidermal tissues, extracellular matrices (ECMs) function as barriers between the organism and environment. Despite being at the interface with the environment, little is known about the role of animal barrier ECMs in sensing stress and communicating with cytoprotective gene pathways in neighboring cells. We and others have identified a putative damage sensor in the *C*. *elegans* cuticle that regulates osmotic, detoxification, and innate immune response genes. This pathway is associated with circumferential collagen bands called annular furrows; mutation or loss of furrow collagens causes constitutive activation of osmotic, detoxification, and innate immune response genes. Here, we performed a genome-wide RNAi screen for modulators of osmotic stress response gene *gpdh-1* in a furrow collagen mutant strain. RNAi of six genes identified in this screen were tested under other conditions and for effects on other stress responses. The functions of these genes suggest negative feedback within osmolyte accumulation pathways and interactions with ATP homeostasis and protein synthesis. Loss of these *gpdh-1* modulators had distinct effects on canonical detoxification and innate immune response genes.

## Introduction

Extracellular matrices (ECMs) are complex networks of secreted fibrous proteins, polysaccharides, and sometimes minerals found in nearly all animal tissues [1]. Traditional functions of ECMs include structural support, tensile strength, elasticity, cell adhesion, and migration [2–4]. It is now clear that ECMs also communicate with cell signaling pathways to influence cell differentiation, tissue morphogenesis, and disease [5–7]. Proteins and growth factors in ECMs interact with cell membrane receptors to regulate migration, differentiation, and tumorigenesis [2,8–10]. Mis-regulation of ECM composition and signaling contributes to arthritis, fibrosis, cancer, and aging [11].

Barrier ECMs are the first line of defense against environmental osmotic pressures, toxins, and pathogens, and are well-positioned to sense environmental stress. Examples include a keratin and lipid-rich epidermis with a collagen foundation in mammals, a rigid chitinous exoskeleton in arthropods, and a flexible collagen-rich cuticle in nematodes [12–15]. Dermal ECM proteins have well-understood roles in transmitting acute mechanical signals to touch sensory neurons in mammals and nematodes [16,17]; alternatively, the role of animal ECMs in sensing environmental stress and regulating cytoprotective gene responses is poorly understood. In fungal cells, several transmembrane sensor proteins detect mechanical stimuli from cell wall damage or cell volume changes and regulate downstream MAPK cascades such as ‘HOG’ (High-Osmolarity Glycerol) to activate cytoprotective and cell wall repair genes [18–20]. Discovery of similar barrier ECM damage sensors in animals would provide new fundamental insights into signaling of stress responses that influence health and distributions of wild populations and degenerative diseases, aging, and longevity in humans [21–24].

The cuticle of nematodes is composed largely of collagen [12,25]. Between 14–16 years ago, genome-wide genetic screens made the surprising discovery that loss of a few cuticle collagen genes strongly activated *C*. *elegans* osmotic and innate immune response genes in the absence of stress [22,26–28]; these results suggested that the cuticle may be involved in sensing environmental stress and regulating relevant cytoprotective genes in neighboring cells. A mucin-like protein secreted by epidermal cells also negatively regulates these same cytoprotective genes further implicating the presence of an extracellular stress sensor [29,30]. To investigate the nature of this putative cuticle sensor and test if other stress responses were affected, we used RNAi to screen 40 genes that are required for diverse aspects of cuticle and epidermal integrity for activation of six core stress responses [31]. Dumpy *‘dpy’* is a class of 26 genes, most encoding cuticle collagens, that all cause a short and wide body shape when mutated or silenced. We discovered that loss of any one of a subset of six *dpy* genes (*dpy-2*, *3*, *7*, *8*, *9*, or *10*) activates broad osmotic, innate immune, and detoxification stress responses without activating heat shock or organelle-specific stress responses [31]. Loss or mutation of any one of these six genes disrupts organization of circumferential bands of collagen termed ‘annular furrows’ and at least one encodes a collagen localized to furrows [31–34]. These results are consistent with furrows being part of, or interacting with, a sensor for cuticle damage that regulates three environmental stress responses related to barrier defenses.

To learn more about processes that influence this putative ECM damage signal, we used a dsRNA-expressing *E*. *coli* library to screen the *C*. *elegans* genome for genes that modify fluorescence of a reporter for canonical osmotic stress response gene *gpdh-1*; GPDH-1 is an enzyme for synthesis of glycerol [35]. Secondary assays and RT-qPCR verified *gpdh-1* suppression in *dpy-7* mutants by RNAi of two genes that function in ATP homeostasis; *gpdh-1* expression was enhanced by RNAi of genes for tRNA ligases and organic osmolyte accumulation enzymes. RNAi of these genes had weak or opposite effects on expression of canonical detoxification and innate immune stress response genes suggesting specificity to the osmotic stress response. These results are consistent with glycerol synthesis being linked to energy balance, protein translation acting parallel to furrow disruption, and negative feedback within and between osmolyte accumulation pathways.

## Materials and methods

### *C*. *elegans* strains

All *C*. *elegans* strains were cultured at 20°C using standard methods [36]. The following strains were used: N2 Bristol, CB88 *dpy-7(e88)* X, and QV261 *dpy-7(e88); kbIs24[gpdh-1p*::*dsRed2; myo-2p*::*GFP; pDP#MM051(unc-119*(+))]. The *gpdh-1p*::*dsRed2* reporter contains the same genomic DNA region as a GFP reporter used in prior studies [26]; it starts 3 kb upstream from the *gpdh-1* start codon and is fused to *dsRed2* at the beginning of exon 2.

### RNAi screen

A genome-wide RNAi screen was performed by feeding worms *Escherichia coli* [HT115(DE3)] engineered to synthesize double-stranded RNA (dsRNA). RNAi clones were taken from the ORFeome RNAi feeding library (Open Biosystems, Huntsville, AL) and missing clones were supplemented from the MRC genomic library (Geneservice, Cambridge, UK). Bacteria clones were grown in 200 μl of LB broth with selective antibiotic using standard methods and washed in liquid NGM buffer [37]. Embryos from QV261 worms were collected by bleach synchronization and 20–30 were added to each well of 96-well plates containing bacteria in NGM buffer with 25 μg/ml carbenicillin and 3 mM isopropyl β-d-1-thiogalactopyranoside (IPTG). When worms reached the young adult stage, they were screened manually for fluorescence with a Zeiss Discovery V12 Stereo microscope. Fluorescence was compared to worms fed control dsRNA clone pPD129.36 (LH4440) encoding a 202-bp dsRNA that is not homologous to any *C*. *elegans* gene; dsRNA clones were scored as candidate *gpdh-1* modulators if dsRed2 fluorescence was either enhanced (greater fluorescence) or suppressed (less fluorescence) in at least 80% of worms in a well relative to wells containing worms that were fed control dsRNA clones.

### RT-qPCR

RNAi agar plates contained dsRNA-expressing bacteria on NGM agar supplemented with 25 μg/ml carbenicillin and 3 mM IPTG. Worms were synchronized with bleach, placed on plates, and grown until the first day of adulthood. Some dsRNA clones were diluted with control dsRNA to avoid developmental arrest as follows: 1/2 *atp-4*, 1/2 *pfk-1*.*1*, 1/4 *hars-1*, and 1/8 *rars-1*. RT-qPCR assays were carried out as described previously using the delta-delta Ct method with some modifications [38]. Each replicate contained 7–15 young adult worms; lysates were treated with dsDNAse for 5 minutes at 37°C (ThermoFisher Scientific EN0771) before reverse transcription (Promega A5001). All reactions were performed in 10 μL volumes in a Realplex^2^ (Eppendorf). Relative expression was normalized to wild type worms on standard NGM agar using the reference gene *rpl-2*. Primers used for RT-qPCR are in S1 Table.

### Microscopy

For fluorescence, worms were mounted on 2% agarose pads with 5 mM levamisole and imaged using an Olympus BX60 microscope with a Zeiss AxioCam MRm camera; GFP or RFP filters were used for fluorescence. Exposure settings were consistent for RFP across all conditions, and adjustments to contrast and brightness were made equally across conditions and evenly to whole images. Color was added and merged using ImageJ Version 1.53c. Furrows were imaged by differential interference contrast (DIC) on the same microscope. Body shape was measured as described previously [34].

### Glycerol and motility assays

Glycerol assays were conducted on populations of worms grown on agar as described for RT-qPCR. Samples were lysed by sonication and processed as described previously using the PicoProbe Free Glycerol Fluorometric Assay Kit (Biovision K643-100) [31,39]. Values were normalized to total protein using the Pierce BCA Protein Assay Kit (ThermoFisher Scientific 23227). For acute motility assays, worms were transferred to high NaCl agar by chunking and motility was scored as described previously [39].

### Statistical analysis

Relative mRNA means were normalized to means for controls, log2 transformed, and then compared to a value of 1.0 with one-sample t-tests using Benjamini-Hochberg corrections for multiple comparisons. Glycerol levels were compared to control conditions using a one-way ANOVA with Dunnett post-hoc tests. *p*-values < 0.05 were taken as significant. Data was graphed with Prism 5.04 (La Jolla, CA). Gene ontology enrichment of genes was identified with DAVID functional analysis, and we report Benjamini adjusted *P*-values [40].

## Results and conclusions

### Identification of gpdh-1 modulators in a furrow collagen mutant

**Fig 1:**
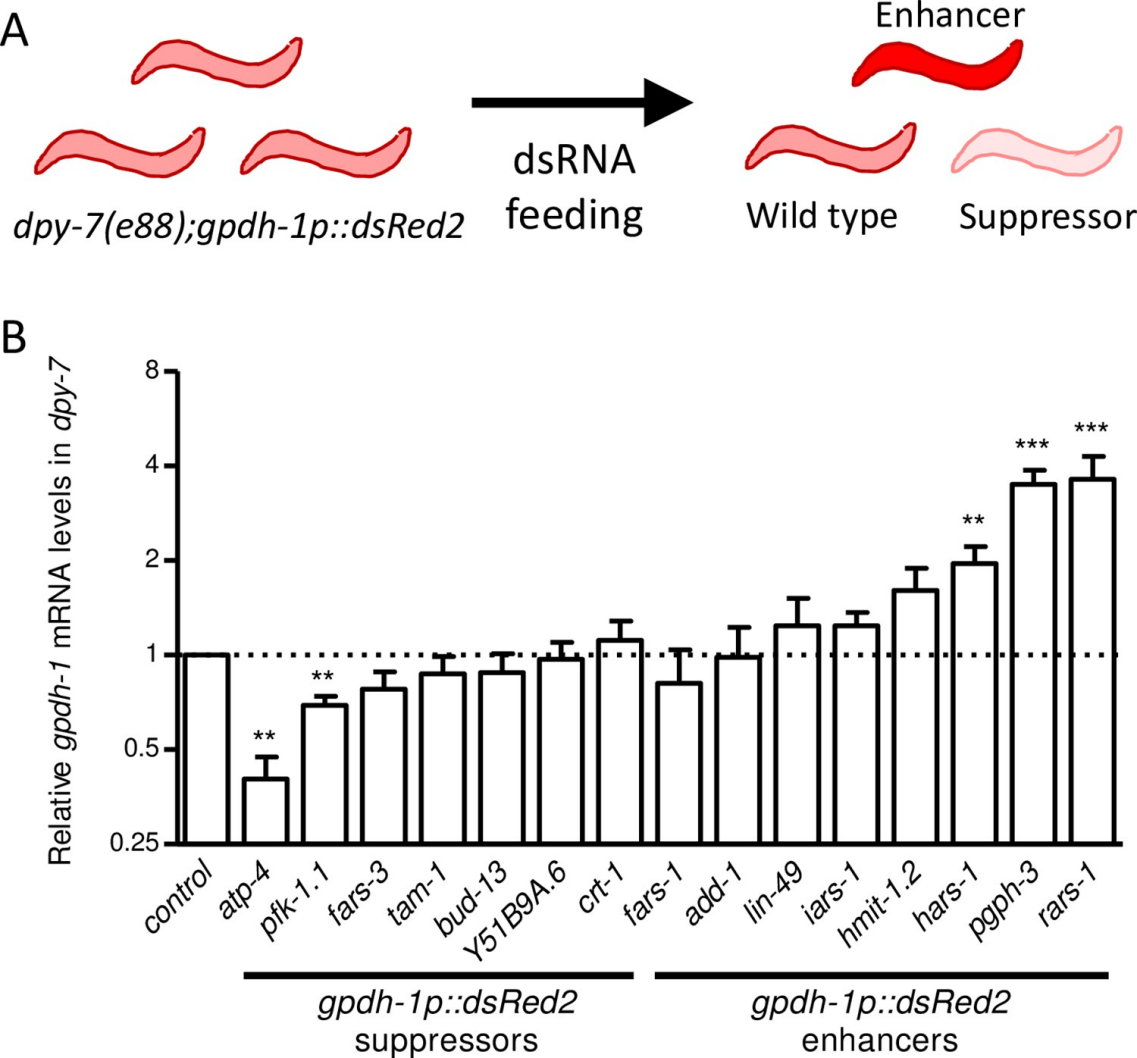
Whole-genome screening identified modulators of *gpdh-1* in *dpy-7* worms. (A) Schematic model of dsRNA screening and scoring of *dpy-7(e88);gpdh-1p*::*dsRed2* worms. (B) Mean plus standard error of relative *gpdh-1* mRNA levels in worms fed dsRNA expressing bacteria that changed *gpdh-1p*::*dsRed2* fluorescence. N = 3 to 12 populations of worms, *P < 0.05, **P < 0.01, ***P < 0.001 determined by one-sample *t-test* with multiple comparisons corrections versus a normalized value of 1.0.

During hyperosmotic stress and in furrow collagen mutants, *C*. *elegans* accumulates the organic osmolyte glycerol in part by inducing *gpdh-1* [26,35]; *gpdh-1* encodes one of two *C*. *elegans* glycerol-3-phosphate dehydrogenase enzymes and is constitutively induced in furrow collagen mutants [26,31]. Fluorescent reporters driven by the *gpdh-1* promoter correlate well with *gpdh-1* mRNA levels [26,31]. In an initial genome-wide screen, 31 clones enhanced, and 242 clones suppressed *gpdh-1p*::*dsRed2* fluorescence in *dpy-7(e88)* worms (S2 Table and Fig 1A). After rescreening these candidates and verifying plasmid sequences, there were eight confirmed dsRNA clones that enhanced, and seven that suppressed, *gpdh-1p*::*dsRed2* fluorescence in at least two out of three trials (S3 Table).

Using RT-qPCR in a *dpy-7* background, two suppressor and three enhancer dsRNA clones were confirmed to significantly affect *gpdh-1* mRNA levels in the directions expected based on reporter scoring (Fig 1B); another clone, *hmit-1*.*2*, caused a 60% increase that was near the significance threshold when adjusted for repeated measures (adjusted *p* = 0.098). The failure of other dsRNA clones to affect *gpdh-1* mRNA in these assays could be caused by inconsistent RNAi effects or transgene array expression modification (e.g., *tam-1* and *add-1*) [41,42]; it is also possible that some act post-transcriptionally because the *gpdh-1p*::*dsRed2* reporter used for screening includes some coding sequence and O*-*GlcNAc transferase OGT-1 was recently shown to regulate GPDH-1 protein [43]. Images of *gpdh-1p*::*dsRed2* fluorescence in *dpy-7* worms fed *atp-4*, *pfk-1*.*1*, *hmit-1*.*2*, *hars-1*, *pgph-3*, or *rars-1* dsRNA clones are shown in S1 Fig; relative *gpdh-1p*::*dsRed2* fluorescence generally matched relative *gpdh-1* mRNA changes. Loss of furrows from the cuticle can be observed with high magnification DIC [34,44]; as shown in S2 Fig, furrows were not restored by any of these dsRNA clones. Furthermore, the associated Dpy (short and wide) body shape phenotype [34] was not suppressed or enhanced by any of these dsRNA clones (S3 Fig).

The *gpdh-1* modulators can be grouped into three functional categories: ATP homeostasis (*atp-4* or *pfk-1*.*1*), protein synthesis (*rars-1* or *hars-1*), and osmolyte accumulation (*pgph-3* or *hmit-1*.*2*). ATP-4 is a homolog of mitochondrial coupling factor 6 and a subunit of ATP synthase, and PFK-1.1 is core glycolysis enzyme phosphofructokinase [45]. RARS-1 and HARS-1 are aminoacyl-tRNA ligases that link tRNAs to arginine and histidine, respectively, for protein translation [46]. PGPH-3 is a glycerol-3-phosphate phosphatase and catalyzes the final step of glycerol synthesis from a glycolysis intermediate. HMIT-1.2 is a homolog of H^+^/myo-inositol transporters that is induced by osmotic stress and promotes viability in worms cultured in high concentrations of NaCl [47].

### Effects of gpdh-1 modulators vary with condition

**Fig 2:**
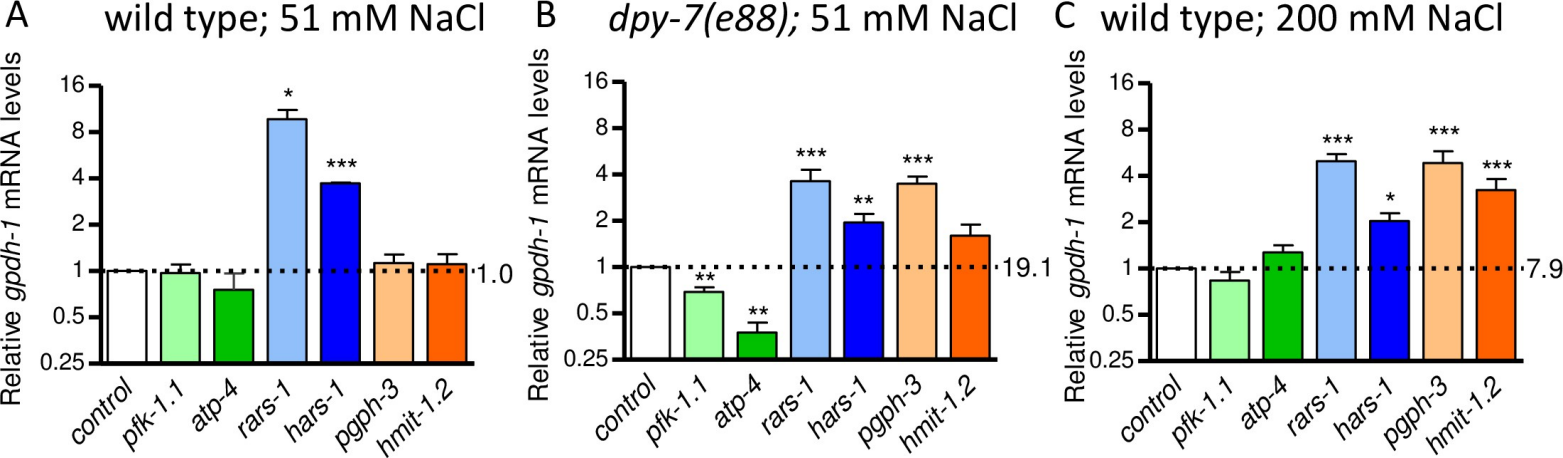
Effects of *gpdh-1* modulators vary with condition. Mean plus standard error of relative *gpdh-1* mRNA levels in worms fed dsRNA expressing bacteria in wild type (A), *dpy-7(e88)* worms on 51 mM (replotted from Fig 1B) (B), and wild type worms on 200 mM NaCl agar (C). The control RNAi worms in *dpy-7* (B) and 200 mM NaCl (C) had *gpdh-1* mRNA levels that were elevated 19.1 and 7.9-fold relative to wild type 51 mM controls, respectively. N = 3 to 12 populations of worms, *P < 0.05, **P < 0.01, ***P < 0.001 determined by one-sample *t-test* with multiple comparisons corrections versus a normalized value of 1.0.

Our RNAi screen and results in Fig 1B are from *dpy-7* worms with disrupted furrows. Exposure to high osmolarity activates many of the same stress response genes as furrow disruption but also likely involves other damage signals including impaired protein translation [26,31,48,49]. We next tested silencing of *gpdh-1* modulators in wild type worms under basal (51 mM NaCl) and mild hyperosmotic conditions (200 mM NaCl) (Fig 2). To make comparisons simple and to highlight the effects of RNAi within each condition, data in each panel of Fig 2 are normalized to control RNAi within each condition and results for *dpy-7* worms are replotted from Fig 1B. Relative to the basal condition (wild type at 51 mM NaCl), *gpdh-1* mRNA levels were induced 19.10 and 7.91-fold in *dpy-7* and 200 mM NaCl worms, respectively (Fig 2B and 2C).

RNAi of *atp-4* or *pfk-1*.*1* only reduced *gpdh-1* expression in *dpy-7* worms with no effect under basal or hyperosmotic conditions suggesting that their effects are specific to worms with disrupted furrows (Fig 2). Hyperosmotic stress decreases protein translation [49]. Loss of aminoacyl-tRNA ligases decreases protein translation and increases *gpdh-1* expression in wild type worms under basal conditions; these results are consistent with a model in which disruption of protein synthesis can act as a signal for osmotic stress [26,49]. As shown in Fig 2, RNAi of either aminoacyl-tRNA ligase gene *rars-1* or *hars-1* increased *gpdh-1* expression under all three conditions consistent with translation inhibition being at least partially additive to furrow disruption and mild hyperosmolarity. RNAi of either osmolyte accumulation gene *pgph-3* or *hmit-1*.*2* increased *gpdh-1* expression in 200 mM NaCl but had no effect on wild type worms in 51 mM NaCl (Fig 2A and 2C). These results suggest that loss of osmolyte accumulation genes reduces negative feedback onto *gpdh-1* expression under conditions that normally activate hyperosmotic stress responses.

**Fig 3:**
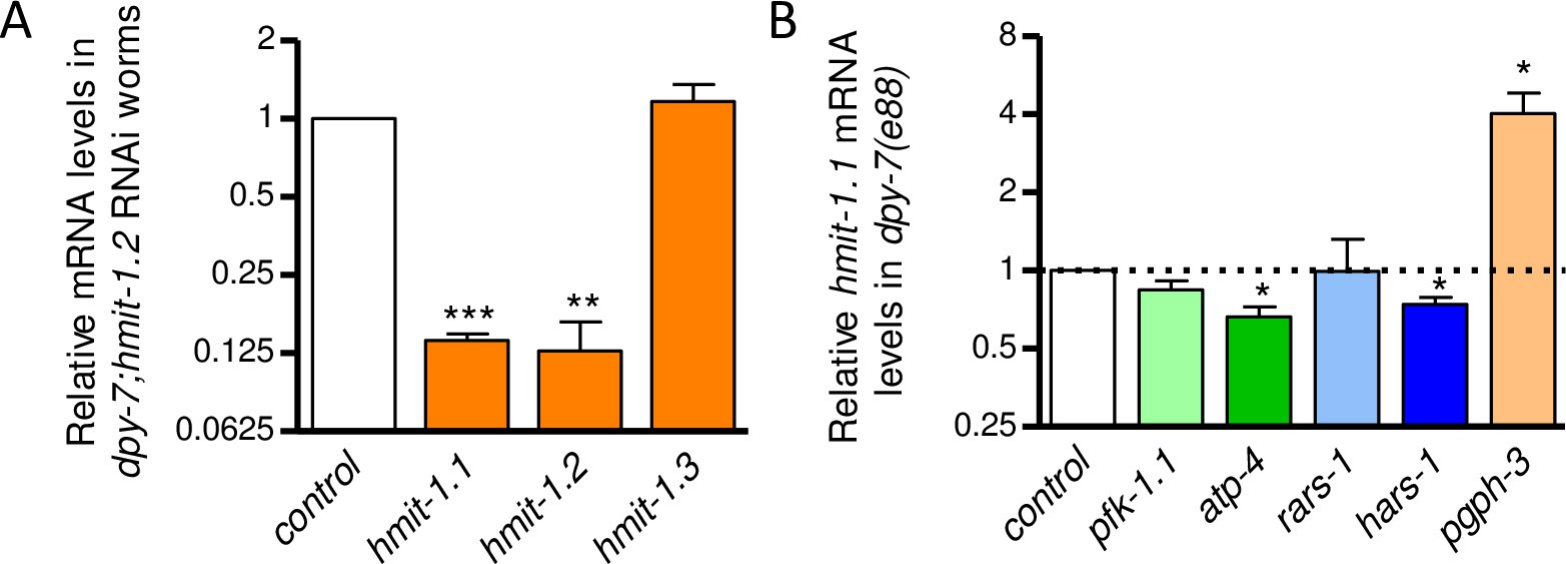
Glycerol and myo-inositol accumulation pathways interact *via* feedback. (A) Mean plus standard error of relative *hmit-1*.*1*, *hmit-1*.*2*, and *hmit-1*.*3* mRNA levels in *dpy-7(e88)* worms fed dsRNA for *hmit-1*.*2*. N = 4 populations of worms. (B) Mean plus standard error of relative *hmit-1*.*1* mRNA levels in *dpy-7(e88)* worms fed dsRNA of *gpdh-1* modulators. N = 6 to 7 populations of worms, *P < 0.05, **P < 0.01, ***P < 0.001 determined by one-sample *t-test* with multiple comparisons corrections versus a normalized value of 1.0.

Enhancement of *gpdh-1* expression by *hmit-1*.*2* RNAi suggests crosstalk between myo-inositol and glycerol accumulation pathways. *C*. *elegans* have three *hmit* paralogs that are expressed in different tissues; *hmit-1*.*1* and *hmit-1*.*2* are recent gene duplicates with many regions of nearly identical nucleotide coding sequence [47]. As shown in Fig 3A, the *hmit-1*.*2* dsRNA clone targets both *hmit-1*.*1* and *hmit-1*.*2* mRNA without affecting *hmit-1*.*3*. *hmit-1*.*1* is expressed in the intestine with *gpdh-1* and is the paralog mostly strongly induced by hyperosmotic stress and loss of furrow collagens [26,31,47]. Fig 3B shows effects of *gpdh-1* modulator RNAi on *hmit-1*.*1* expression; relative to wild type worms, *hmit-1*.*1* mRNA levels were induced 205-fold in *dpy-7* worms. RNAi of *pgph-3* further enhanced *hmit-1*.*1* mRNA 4-fold in *dpy-7* worms; together with data in Fig 2B, this result suggests that crosstalk between osmolyte accumulation pathways occurs in both directions.

RNAi of *pfk-1*.*1* had no effect on *hmit-1*.*1* mRNA (Fig 3B). Unlike their strong enhancing effect on *gpdh-1* mRNA (Fig 2), neither *rars-1* nor *hars-1* RNAi increased *hmit-1*.*1* mRNA suggesting that reduced protein synthesis may not activate myo-inositol accumulation mechanisms.

### RNAi of rars-1 or hars-1 increases glycerol accumulation in dpy-7 worms

**Fig 4:**
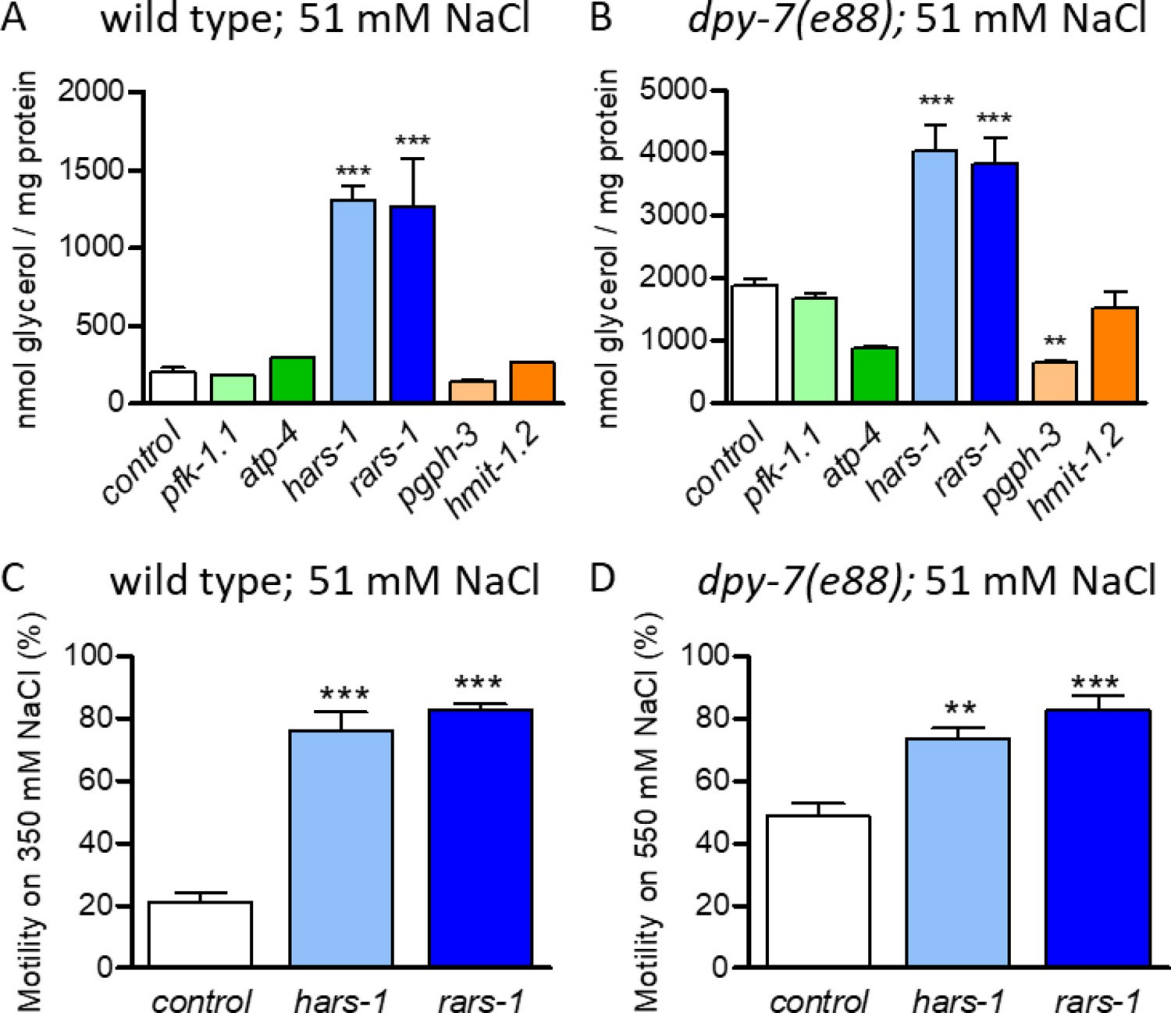
*gpdh-1* modulators affect glycerol accumulation. Mean plus standard error of whole-worm glycerol levels in wild type (A) and *dpy-7(e88)* worms (B) fed dsRNA of *gpdh-1* modulators. (A and B) N = 3 to 7 populations of worms, *P < 0.05, **P < 0.01, ***P < 0.001 determined by one-way ANOVA with a Dunnett post-hoc test. (C and D) Motility of worms 20 minutes after transfer to agar containing 350 (C) or 550 mM NaCl (D); N = 6 to 10 populations of worms. (A-D) *P < 0.05, **P < 0.01, ***P < 0.001 determined by one-way ANOVA with a Dunnett post-hoc test.

Given that *gpdh-1* encodes a rate-limiting enzyme for glycerol synthesis and that furrow collagen mutant worms accumulate high levels of glycerol, we next tested if the *gpdh-1* modulators we identified affect accumulation of glycerol in *dpy-7* or wild type worms under basal conditions. In wild type worms on 51 mM NaCl, glycerol levels were not decreased significantly by RNAi of *pgph-3* consistent with residual PGPH activity or other synthesis pathways being sufficient for low basal levels (Fig 4A). Alternatively, RNAi of *pgph-3* decreased glycerol levels in *dpy-7* worms (Fig 4B). RNAi of *rars-1* or *hars-1* strongly increased glycerol levels in wild type and *dpy-7* worms (Fig 4). We also tested if the strong *gpdh-1* effects of *rars-1* or *hars-1* RNAi would be reflected in motility after transfer to high motility. As shown in Fig 4C and 4D, *rars-1* and *hars-1* RNAi increased acute motility on high NaCl under both conditions.

RNAi of neither *hmit-1*.*2* nor *pfk-1*.*1* significantly changed glycerol levels consistent with small effects on *gpdh-1* mRNA levels (Figs 2 and 4). There was a trend of reduced glycerol levels with RNAi of *atp-4* in *dpy-7* worms, but this effect did not reach the threshold for statistical significance (*p* = 0.0997) (Fig 4B).

### gpdh-1 modulators are stress response gene specific

**Fig 5:**
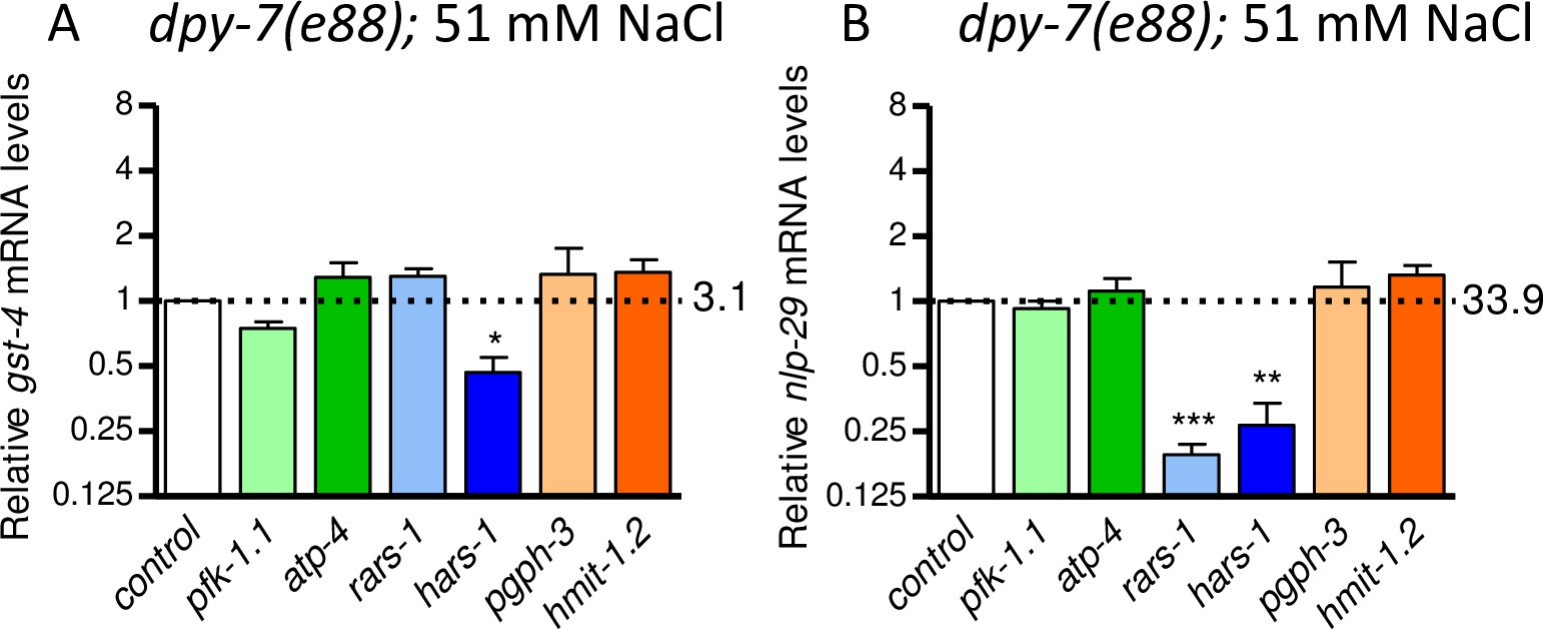
*gpdh-1* modulators are stress response specific. Mean plus standard error of relative *gst-4* (A) and *nlp-29* (B) mRNA levels in *dpy-7*(e88) worms fed dsRNA of *gpdh-1* modulators. N = 4 to 8 populations of worms, *P < 0.05, **P < 0.01, ***P < 0.001 determined by one-sample *t-test* with multiple comparisons corrections versus a normalized value of 1.0.

Lastly, we tested the effects of furrow-sensitive *gpdh-1* modulators on core detoxification (*gst-4*) and innate immune response genes (*nlp-29*) [31,50–53]. Relative to wild type worms on 51 mM NaCl, *gst-4* and *nlp-29* mRNA levels were induced 3.1 and 33.9-fold in *dpy-7;control RNAi* worms, respectively (Fig 5). Compared to effects on *gpdh-1* (Fig 2B), RNAi of *gpdh-1* modulators had reduced or opposite effects on *gst-4* and *nlp-29* mRNA (Fig 5). For example, RNAi of *pfk-1*.*1*, *atp-4*, *pgph-3*, and *hmit-1*.*2* had no effect on either *gst-4* or *nlp-29*. RNAi of *rars-1* suppressed expression of *nlp-29* and RNAi of *hars-1* suppressed expression of *gst-4* and *nlp-29*. These data suggest that the effects of aminoacyl-tRNA ligase, ATP homeostasis, and osmolyte accumulation gene silencing on *gpdh-1* are not shared with other stress response genes activated by furrow disruption.

## Discussion

Defining interactions between extracellular matrix sensors and cellular stress responses is expected to reveal novel signaling mechanisms. Unfortunately, our RNAi screen did not identify any genes with obvious signaling functions. False negative rates are high with large-scale *C*. *elegans* RNAi screens [54]; relevant signaling genes might have redundancy, could be missing from our RNAi library, or might cause lethality when silenced. From a targeted screen of 381 *C*. *elegans* predicted protein kinase genes, we recently identified a membrane kinase named DRL-1 that is required for full activation of osmotic, detoxification, and innate immune responses in *dpy-7* mutant worms [39]. Although our current genome-wide screen did not identify genes likely to function directly in activation of stress responses, the genes identified reveal new insights into how osmotic stress responses are regulated by negative feedback and integrated with ATP homeostasis and protein synthesis signaling.

### Disruption of ATP homeostasis genes restricts gpdh-1 activation

Glycerol metabolism is tightly linked to carbohydrate homeostasis and is central to lipid synthesis [55]. Loss of ATP-4 or PFK-1.1 would be expected to disrupt ATP homeostasis. Suppression of *gpdh-1* mRNA by RNAi of *atp-4* or *pfk-1*.*1* suggests that the osmotic stress response is restricted when ATP homeostasis is disrupted in furrow collagen mutant worms. RNAi of *pfk-1*.*1* and *atp-4* had little or no effect on expression of detoxification and innate immune response genes *gst-4* and *nlp-29*, respectively (Fig 5) suggesting that the effect of ATP homeostasis gene silencing is specific to the osmotic stress response. Surprisingly, RNAi of *atp-4* or *pfk-1*.*1* did not suppress *gpdh-1* expression in worms exposed to 200 mM NaCl (Fig 2C) suggesting that interactions between *gpdh-1* regulation and energy metabolism during mild hyperosmotic stress might be different than when furrows are disrupted.

A previous study conducted a similar RNAi screen for suppressors of *gpdh-1* in *osm-8* mutants [30]; OSM-8 encodes a protein secreted by epidermal cells with homology to mucins and is predicted to function downstream from furrow collagens [30,56]. *osm-8* mutants activate the same stress responses as furrow collagen mutants with no obvious defects in cuticle organization [29,30,44]. Analysis of genes required for *gpdh-1* expression in *osm-8* worms identifies oxidative phosphorylation as the most enriched functional category [30,40] (S4 Fig). Therefore, ATP homeostasis may be a common requirement for full *gpdh-1* activation in *osm-8* and furrow collagen mutant worms. Another gene required for *gpdh-1* activation in *osm-8* worms is *ptr-23*, which encodes an uncharacterized protein with predicted transmembrane domains [30]. Like our results for *pfk-1*.*1* and *atp-4*, *ptr-23* was not required for *gpdh-1* activation by high salt [30]. Future studies are needed to determine if *ptr-23* functions in furrow collagen mutants.

### Depletion of aminoacyl-tRNA ligases and furrow disruption initiate distinct responses

Protein misfolding and aggregation occur rapidly and broadly during hyperosmotic stress in *C*. *elegans* [22,48,57,58] and translation of new protein is suppressed to attenuate this damage [49,59]. Inhibition of translation pharmacologically or by depletion of aminoacyl-tRNA ligases activates *gpdh-1* expression supporting a model in which uncharged tRNAs can act as a signal for hyperosmotic stress [26,49]. Depletion of aminoacyl-tRNA ligases activated *gpdh-1* and glycerol accumulation additively with furrow collagen gene mutation. This result could be explained by furrow disruption only partially reducing aminoacyl-tRNA ligation or by furrow loss and uncharged tRNAs initiating different signaling pathways that converge on *gpdh-1*. Disruption of furrows activates canonical detoxification (*gst-4*) and innate immune response (*nlp-29*) genes together with *gpdh-1* and *hmit-1*.*1* [29,31]. Depletion of aminoacyl-tRNA ligases either had no strong effect, or suppressed, *hmit-1*.*1*, *gst-4*, and *nlp-29* (Figs 3 and 5) consistent with distinct cytoprotective responses to uncharged tRNAs and furrow disruption. Alternatively, we and others have demonstrated striking correlation between genome-wide transcriptome changes caused by hyperosmolarity and mutation of furrow collagens [29,31].

### Negative feedback within and between osmolyte accumulation pathways

Glycerol is synthesized from glycolysis intermediate metabolite dihydroxyacetone phosphate in two steps, the first reaction is catalyzed by GPDH and the second by PGPH. RNAi of *pgph-3* lowered glycerol levels and enhanced *gpdh-1* expression under conditions that normally induce the osmotic stress response. These results are consistent with a negative feedback loop where glycerol accumulates to an adjustable set-point and represses further *gpdh-1* expression. Strong induction of *hmit-1*.*1* by high osmolarity and furrow loss suggests that myo-inositol also has a role [31,34,47]. Under conditions that induce osmotic stress responses, RNAi of *hmit-1*.*1*/*1*.*2* enhanced *gpdh-1* expression and RNAi of *pgph-3* enhanced *hmit-1*.*1* expression suggesting that a feedback mechanism is sensitive to total internal osmolarity contributed by both glycerol and myo-inositol and represses expression of genes that promote accumulation of both osmolytes. RNAi of *pgph-3* or *hmit-1*.*1/1*.*2* had no effect on canonical detoxification (*gst-4)* and innate immune response genes (*nlp-29*) consistent with a feedback mechanism that is specific to osmotic responses.

Nematodes maintain a positive internal turgor pressure against the cuticle that functions as a hydrostatic skeleton; in large species that allow direct measurements, this turgor pressure was found to be 10–70 mmHg [60–63]. Maintenance of turgor pressure is required for motility and would be expected to be under tight regulation. Glycerol and myo-inositol are organic osmolytes compatible with protein folding that are expected to contribute to turgor pressure and the hydrostatic skeleton. Mechanical strain between the cuticle and underlying tissues is one possible mechanism for sensing turgor pressure. Exposure to high osmolarity environments causes osmotic water loss and reduced turgor pressure that could initiate a mechanical signal for stress response activation [22,63–65]. Mutation or loss of furrow collagens may mimic mechanical changes caused by hyperosmotic water loss by relaxing mechanical strain on turgor pressure sensors [66–68].

## Supporting information

S1 Fig Paired bright field and merged fluorescent images of QV261 worms fed dsRNA of *gpdh-1* modulators. Note that exposure settings are the same for all fluorescent images resulting in saturation of the *rars-4* RNAi worms. Scale bar is 200 microns.
(TIF)

S2 Fig Representative DIC images of cuticles in *dpy-7* worms fed dsRNA of *gpdh-1* modulators. Furrows are lost in *dpy-7* adults fed control or *gpdh-1* modulator dsRNA clones. Scale bar is 25 microns.
(TIF)

S3 Fig RNAi of *gpdh-1* modulators does not change body shape. N = 15 individual worms per condition.
(TIF)

S4 Fig ATP homeostasis genes are required for *gpdh-1* activation in *osm-8* mutants. Genes identified in a prior RNAi screen for regulation of *gpdh-1* in *osm-8* mutants [30] were analyzed for gene ontology enrichment with DAVID [40].
(TIF)

S1 Table Primer sequences. (XLSX)

S2 Table dsRNA gene hits. (XLSX)

S3 Table Confirmed dsRNA genes. (XLSX)
