# Supplementary figures and images for: RNAi screening for modulators of an osmo-sensitive gene response to extracellular matrix damage reveals negative feedback and interactions with translation inhibition
Source: PLoS One. 2023 May 8;18(5):e0285328. doi: 10.1371/journal.pone.0285328 (PMC10166495; doi:10.1371/journal.pone.0285328)

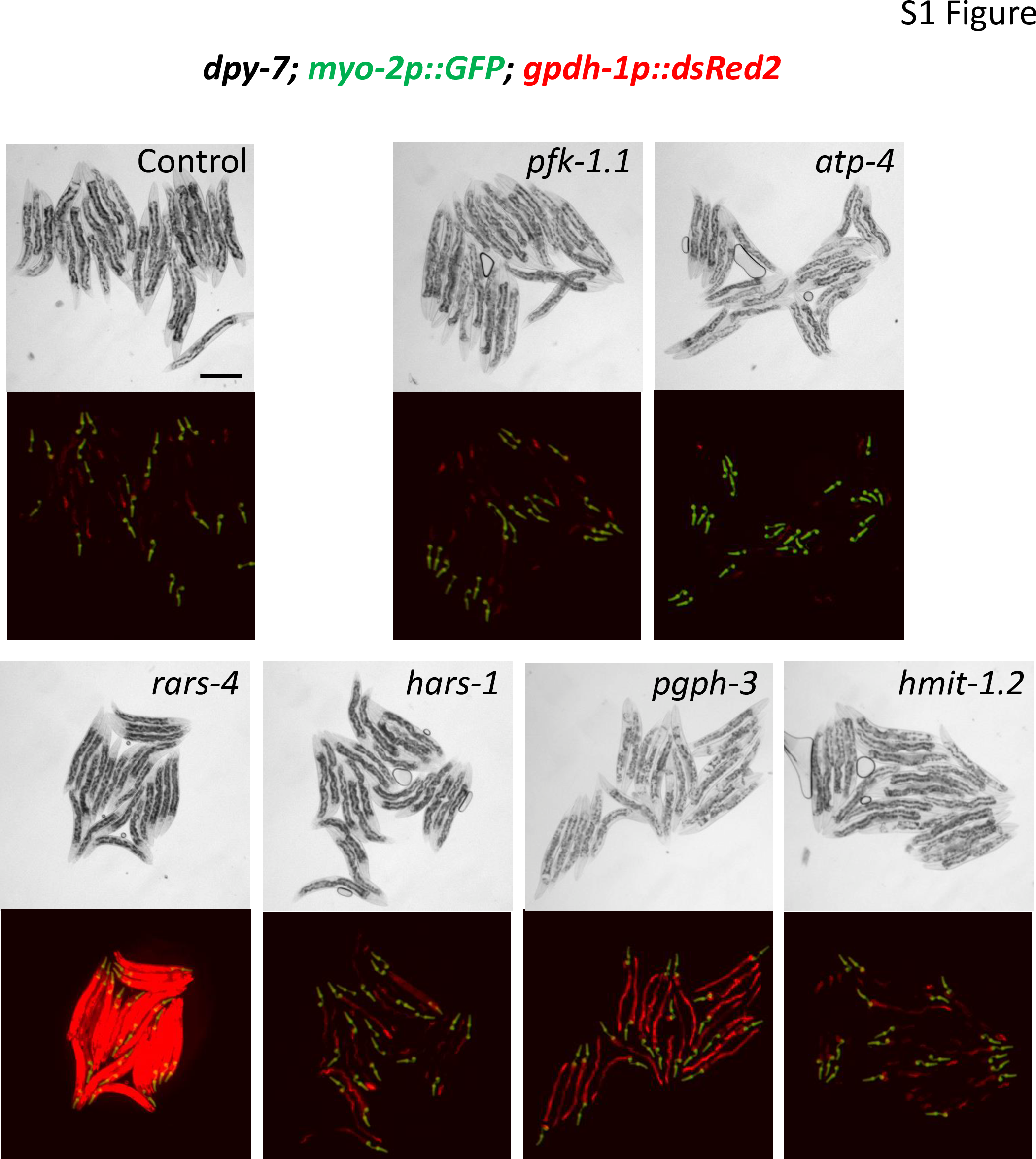

Supplement: S1 Fig — Note that exposure settings are the same for all fluorescent images resulting in saturation of the rars-4 RNAi worms. Scale bar is 200 microns. (TIF) [file pone.0285328.s001.tif]

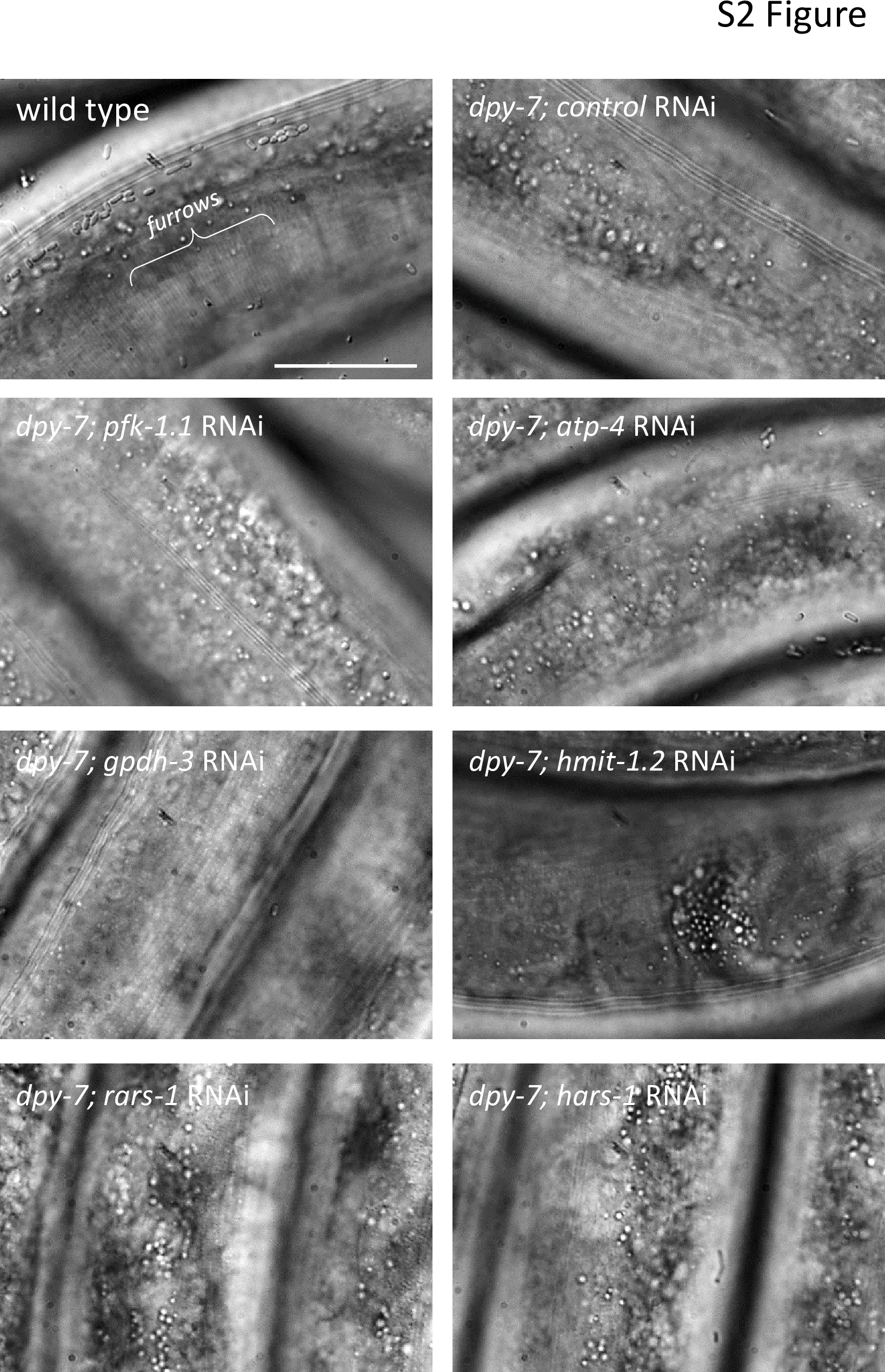

Supplement: S2 Fig — Furrows are lost in dpy-7 adults fed control or gpdh-1 modulator dsRNA clones. Scale bar is 25 microns. (TIF) [file pone.0285328.s002.tif]

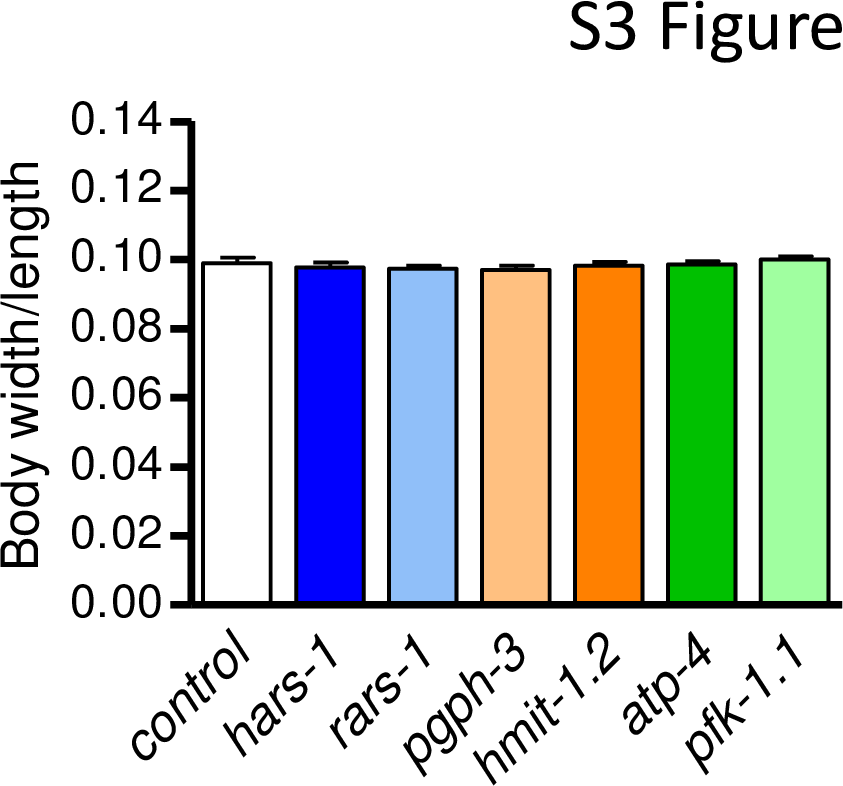

Supplement: S3 Fig — N = 15 individual worms per condition. (TIF) [file pone.0285328.s003.tif]

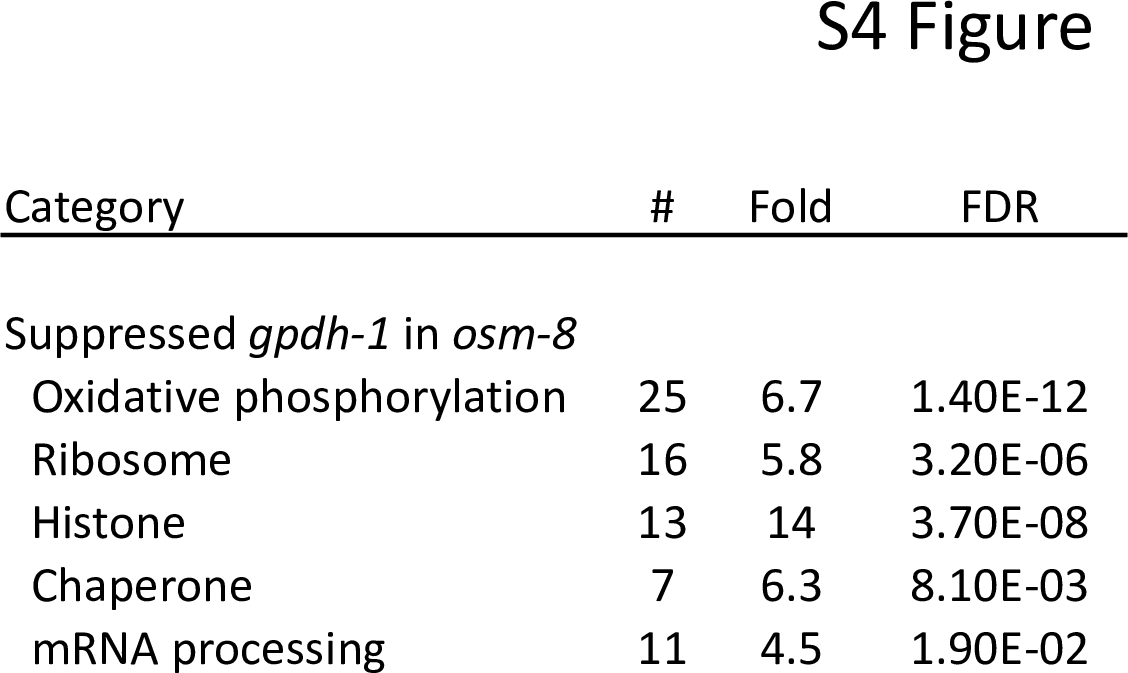

Supplement: S4 Fig — Genes identified in a prior RNAi screen for regulation of gpdh-1 in osm-8 mutants [30] were analyzed for gene ontology enrichment with DAVID [40]. (TIF) [file pone.0285328.s004.tif]
